# Genome-Wide Association Studies Prioritize Genes Controlling Seed Size and Reproductive Period Length in Soybean

**DOI:** 10.3390/plants13050615

**Published:** 2024-02-23

**Authors:** Le Wang, Fu’an Niu, Jinshe Wang, Hengyou Zhang, Dan Zhang, Zhenbin Hu

**Affiliations:** 1 State Key Laboratory of Black Soils Conservation and Utilization, Key Laboratory of Soybean Molecular Design Breeding, Northeast Institute of Geography and Agroecology, Chinese Academy of Sciences, Harbin 150081, China; wangl@iga.ac.cn; 2 Animal Genomics and Improvement Laboratory, Agricultural Research Service, U.S. Department of Agriculture, Beltsville, MD 20705, USA; 3 Collaborative Innovation Center of Henan Grain Crops, College of Agronomy, Henan Agricultural University, Zhengzhou 450002, China; 4 Institute of Crop Breeding and Cultivation, Shanghai Academy of Agricultural Sciences, Shanghai 201403, China; niufuan224@126.com; 5 National Innovation Centre for Bio-Breeding Industry, Institute of Crop Molecular Breeding, Henan Academy of Agricultural Sciences, Zhengzhou 450002, China; wjs33314@126.com

**Keywords:** GWAS, soybean, *Glycine max*, 100-seed weight, reproductive period length, *Arabidopsis*

## Abstract

Hundred-seed weight (HSW) and reproductive period length (RPL) are two major agronomic traits critical for soybean production and adaptation. However, both traits are quantitatively controlled by multiple genes that have yet to be comprehensively elucidated due to the lack of major genes; thereby, the genetic basis is largely unknown. In the present study, we conducted comprehensive genome-wide association analyses (GWAS) of HSW and RPL with multiple sets of accessions that were phenotyped across different environments. The large-scale analysis led to the identification of sixty-one and seventy-four significant QTLs for HSW and RPL, respectively. An ortholog-based search analysis prioritized the most promising candidate genes for the QTLs, including nine genes (*TTG2*, *BZR1*, *BRI1*, *ANT*, *KLU*, *EOD1/BB*, *GPA1*, *ABA2*, and *ABI5*) for HSW QTLs and nine genes (such as *AGL8*, *AGL9*, *TOC1*, and *COL4*) and six known soybean flowering time genes (*E2*, *E3*, *E4*, *Tof11*, *Tof12*, and *FT2b*) for RPL QTLs. We also demonstrated that some QTLs were targeted during domestication to drive the artificial selection of both traits towards human-favored traits. Local adaptation likely contributes to the increased genomic diversity of the QTLs underlying RPL. The results provide additional insight into the genetic basis of HSW and RPL and prioritize a valuable resource of candidate genes that merits further investigation to reveal the complex molecular mechanism and facilitate soybean improvement.

## 1. Introduction

Increasing crop grain yield has been a major goal of crop breeding to meet the growing demand for more food in the context of the increasing human population and changing climates. This requires enhanced geographic adaptability and increased seed production for crops. Traditional breeding has greatly contributed to the dramatic increase in grain in staple crops in recent decades, such as wheat, rice, and corn. With the gradual recognition of genetic variation for crop improvement [1,2], mining genomic variants and genes underlying yield-related traits has become increasingly critical for precision crop breeding of high-yield varieties to ensure food supply globally.

Soybean (*Glycine max* (L.) Merr.) was domesticated from wild soybean (*G. soja* Sieb. & Zucc.) in East Asia about 6000–9000 years ago, and many morphological traits have been significantly improved for high production to meet human demand [3,4]. Soybean is, thus far, the most cultivated oil seed crop worldwide because of the richness of high-quality oil (~20%) and protein (~40%) in its seeds [5]. It serves as feedstock and ingredients for diverse industrial uses, animal feed, and human consumption. Because of the highly valuable protein and oil in its seeds, soybeans are regarded as one of the best sources to provide sustainable plant-based protein to feed the world [6]. Although soybean yield (bushels per acre) has nearly doubled since 1987 [7], the increase in soybean production per year cannot keep up with the demand for plant-based protein globally. Therefore, improving soybean yield and production has been a major goal in soybean breeding worldwide. Hundred-seed weight (HSW) and reproductive period length (RPL) are two important agronomic traits that are highly critical for yield and local adaptation [8]. Both traits were reported to be quantitatively controlled for many genes, and they were also reported to be affected by a variety of environmental factors such as photoperiod, temperature, and rainfall [9,10]. Over 300 QTLs have been identified, as indicated in the SoyBase database (https://www.soybase.org/), whereas few of them have been cloned, demonstrating the complexity of the nature of the regulatory mechanism for both traits. It requires continuous efforts to increase our understanding of the underlying mechanisms to facilitate soybean production improvement.

Seed size and flowering time are two phenotypes that have been intensively studied in the model plant species *Arabidopsis*, and many genes participating in a variety of biological pathways have been identified. For seed weight, it has been demonstrated that the ubiquitin–proteasome pathway, G-Protein Signaling (such as AGG3 or AGB1), mitogen-activated protein kinase signaling (such as MKK4/5), brassinosteroids (such as BRI1), auxin (ARF2), and transcriptional regulators (such as TTG2) are involved in its regulation [11]. In soybean, several genes have been cloned to elucidate the mechanisms of seed size control. For example, the *BIG SEEDS1* (*BS1*) gene encodes a transcriptional factor, and the downregulation of *BS1* increased seed size and weight through a regulatory modulation of genes functioning in primary cell proliferation [12]. *PP2C-1* was identified within a large QTL interval, and its function in controlling seed weight was validated in *Arabidopsis* [13]. *CYP78A72* and *CYP78A10* from the cytochrome P450 family genes were associated with seed sizes in soybean and other species, suggesting a conserved seed-size-regulating pathway across species [14,15,16]. *GmST05* (Seed Thickness 05) was uncovered in a GWAS, and it positively regulated seed size [17]. Compared to HSW, RPL determines the success of completing a reproductive period for producing vigorous seeds for the next generation, whereas it has gained much less attention. To the best of our knowledge, several QTLs for RPL have been identified in soybean [18,19]. RPL was found to be negatively correlated with flowering time [19,20]. In contrast, QTL genes controlling flowering time have been well documented in soybean [21,22], such as *E1*, *E2*, *E3*, *E4*, *E9*, *J*, *Tof11*, and *Tof12*, some of which play roles in facilitating local adaptation [23,24,25]. Given the negative correlation between flowering time and RPL, which is important for seed filling, RPL should also be crucial for adaptation and yielding potential. Importantly, many genes controlling flowering and seed size in *Arabidopsis* were also identified in the regulation of HSW or flowering, such as *SWEET39*/*SWEET10a*/*SWEET15* [26,27,28] and *E2*/*GIGANTEA* [29,30]. These and other studies clearly demonstrate the conserved mechanism in the control of flowering time and seed size between the model plant species Arabidopsis and certain crops [31]; therefore, ortholog-based gene cloning would be useful to guide gene discovery in crops.

Considering the recorded QTLs for HSW and RPL (https://www.soybase.org/), there are far more genes yet to be identified in soybean. More efforts are required to identify additional QTL genes to facilitate fundamental research and crop improvement. On the other hand, map-based cloning is time-consuming for pinpointing genes underlying the traits of interest. Genome-wide association studies (GWAS) are useful for identifying genes that are representative in large populations, but nominating the best candidate genes has been challenging mainly due to factors such as large linkage disequilibrium (LD) regions. It is necessary to seek an alternative approach to accelerate the identification of the best candidate genes for the QTLs.

In this study, we conducted large-scale GWAS of HSW and RPL using multiple panels evaluated in different environments. In total, we identified 61 genomic loci (QTLs) for HSW and 74 for RPL. With an ortholog-based gene discovery approach, we nominated eleven soybean orthologs of *Arabidopsis* genes controlling seed size as candidates for 11 HSW QTLs. Additionally, we identified six soybean flowering-time-controlling genes (*E2*, *E3*, *E4*, *Tof11*, *Tof12*, and *GmFT2b*) and 13 soybean orthologs of *Arabidopsis* controlling flowering time and photosensitivity underlying RPL QTLs. Supporting their candidacy, these orthologs were found to be biased towards the corresponding HSW and RPL QTL regions across the genome. We also observed a strong correlation between the genetic diversity of RPL QTLs and geographic distribution. These results greatly enhance our understanding of the genetic basis of these important traits and prioritized candidate genes that could facilitate yield and adaptation improvement in soybean.

## 2. Results

### 2.1. HSW and RPL Exhibit Significant Variations Subjected to Environmental Effects

**Figure 1:**
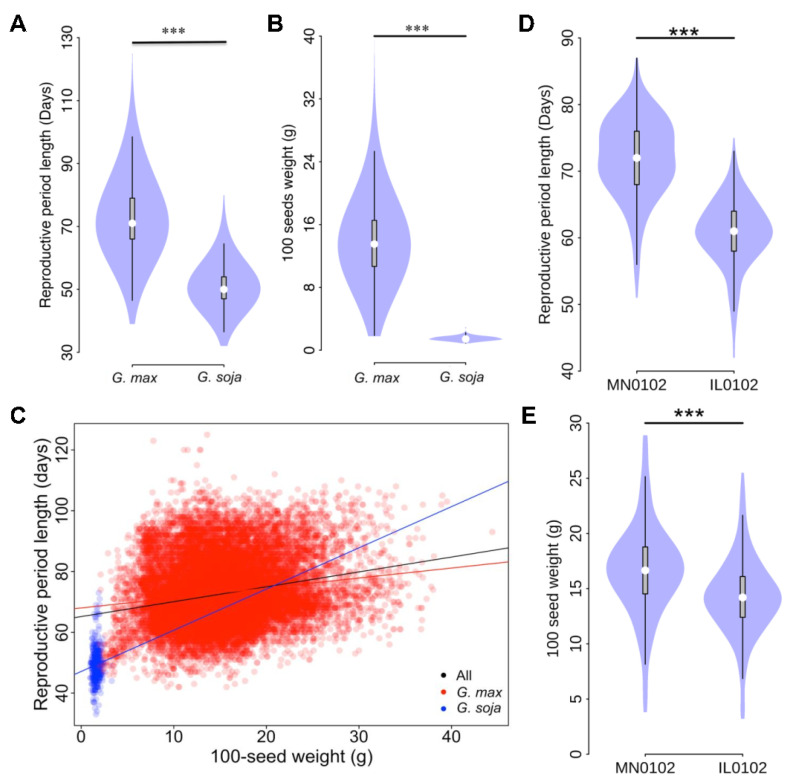
The phenotypic variation in RPL and HSW. (**A**)Comparison between G. soja and G. max for RPL and (**B**) for HSW. (**C**) Correlation between RPL and HSW in soybean population. The G. soja and G. max germplasm are represented with blue and red dots, respectively. (D) Difference of RPL in a soybean panel grown under two environments: MN0102 and IL0102. (**E**) Difference of HSW in a panel under two environments: MN0102 and IL0102. “***” indicates significance at *p* < 0.001.

**Figure 2:**
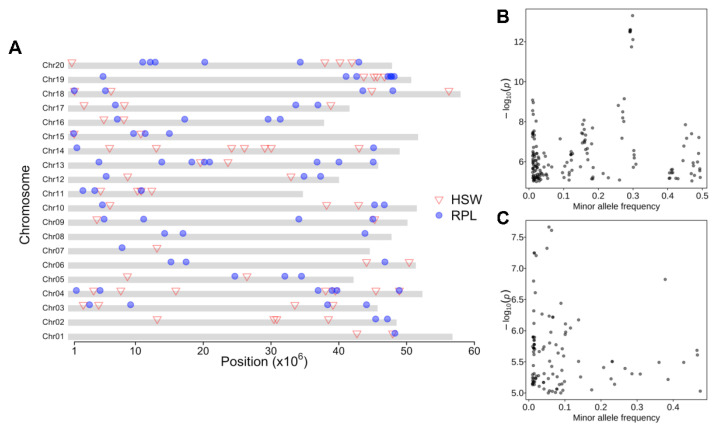
Genome-wide associations for 100-seed weight (HSW) and reproductive period length (RPL). (**A**) Illustration of associations for HSW and RPL across 20 chromosomes. Trait representation symbols for both traits are indicated on the right of the panel. (**B**) Relationship between significant associations and the minor allele frequency for RPL and (**C**) for HSW.

In total, approximately 17,000 observations for HSW and 18,000 observations for RPL were obtained from the Germplasm Resources Information Network (GRIN) database. Both traits exhibited considerable variation (Figure 1A), with RPL ranging from 32 d (e.g., PI522183A and PI562544) to 125 d (e.g., PI561382), with an average of 71 d (Figure 1A). Meanwhile, HSW ranged from 1.0 g (such as PI479748) to 44.4 g (e.g., PI416894), with an average of 15.4 g (Figure 1B). As anticipated, significant differences in (*p* < 10^−16^) in RPL or HSW were observed between *G. max* and *G. soja*, consistent with the fact that both HSW and RPL are subject to human selection during domestication (Figure 1A and Figure 2B). Interestingly, when evaluating RPL–HSW correlation, we observed a significantly positive correlation (*r* = 0.24, *p* < 10^−16^) between RPL and HSW in the *G. max* population (Figure 1C) and in all germplasm (*G. max* and *G. soja*, *r* = 0.16, *p* < 10^−16^). In contrast, no RPL–HSW correlation was seen in *G. soja* (*r* = 0.08, *p* = 0.15) (Figure 1C). To mitigate environmental effects, we also calculated the correlation using datasets from each environment, and the results supported the positive correlation between RPL and HSW (Table S2). This observation suggests that both RPL and HSW were selected toward similar goals in cultivated soybean during domestication and improvement, likely focused on achieving high yield, a trait absent in *G. soja* subpopulation. The heritabilities for HSW and RPL were 0.76 and 0.53, respectively, indicating that both traits were genetically controlled and likely inherited quantitatively due to the phenotypic variation observed.

Since the phenotypes for the accessions were collected across multiple environments, we were interested in exploring whether both traits were influenced by environmental factors. Our analyses revealed significant effects of the environment on both traits (Figure 1). For example, the RPL for accessions grown in Minnesota averaged 71.9 d, whereas the average RPL for the same set of accessions grown in Illinois, south of Minnesota, was approximately 11.2 d shorter, averaging around 60.7 d (Figure 1A). This result indicates that long-day photoperiod tends to prolong the RPL of accessions in the northern areas compared to those in southern areas with shorter photoperiods (Figure 1D). Similarly, significant differences in HSW (*p* < 10^−16^) were also observed for accessions grown in Minnesota and Illinois (Figure 1E). For example, the average HSW for accessions in Minnesota was approximately 16.7 g, which is 2.4 g greater than the average HSW for the same set of accessions in Illinois (14.3 g), strongly suggesting the influence of environmental factors, likely latitude, on HSW (Figure 1E). However, when calculating the correlation between HSW or RPL and latitude, no correlations were observed between HSW and latitude within all germplasm (*r* = 0.008, *p* = 0.92), *G. soja* (*r* = 0.03, *p* = 0.04), or *G. max* (*r* = 0.07, *p* = 5.7 × 10^−9^) (Table S3). In contrast, RPL showed a negative correlation (r = −0.22− −0.14, *p* = 4.6 × 10^−6^ − 2.2 × 10^−16^) with latitude in all germplasm, *G. max*, and *G. soja* subpopulations (Table S3). Surprisingly, we observed moderate correlations between both traits and longitude in all germplasm and subpopulations. For example, HSW–longitude correlations were *r* = 0.27 with *p* < 10^−16^, *r* = 0.30 with *p* = 1.14 × 10^−4^, and *r* = 0.30 with *p* < 10^−16^ in all germplasm, *G. soja*, or *G. max*, respectively; and RPL–longitude correlations were *r* = 0.12 with *p* < 10^−16^, *r* = 0.19 with *p* = 7.3 × 10^−10^, or *r* = 0.24 with *p* < 10^−16^ in all germplasm, *G. soja*, or *G. max*, respectively (Table S3). These results indicate that, although the correlation is not particularly strong, geographic locations have varying effects on HSW and RPL, with relatively larger effects observed in *G. max* compared to *G. soja*.

### 2.2. Large-Scale GWAS Reveals Genome-Wide Associations with HSW and RPL

Given the agronomic importance and observed environmental effects on both HSW and RPL, we conducted GWAS for HSW or RPL using multiple subsets of GRIN germplasm with the phenotypes collected in a single environment (Table S1). Panels containing at least 200 accessions were used in GWAS (Table S1). The dataset allows us to perform GWAS for HSW, with 26 panels consisting of 210 to 1174 accessions, and GWAS for RPL, using 28 panels comprising 210 to 1174 accessions. Applying a defined threshold (*p* value < 10^−5^), we identified 61 QTLs for HSW and 74 QTLs for RPL (Table S5). These QTLs were distributed across all 20 chromosomes, indicating that both traits were quantitatively controlled by multiple genes (Figure 2A; Table S5). Upon closer investigation, we found rare overlap among the identified QTLs for HSW and RPL, suggesting independent control of both traits despite a positive correlation observed between them (Figure 1C). Most QTLs were found to be environment- or population-specific (Figure 2A; Table S5). For example, among the 61 QTLs for HSW, 55 (90.2%) were identified in single environments, with the remaining six (9.8%) detected in at least two environments, such as QTL *C11P11* in both NM832 and MS2001_03, and QTL *C4P16* in both MN0102 and MS2001_02 (Table S5). Similarly, seven (9.4%) of the 74 QTLs for RPL were repeatedly identified in at least two environments, such as QTL *C10P45* in four environments, including 1IL66, 2IL811, IL0102, and IL945, and QTL *C19P47.6* in both 1IL66 and MN0102 (Table S5). Most of the QTLs exhibited small effects, indicating complex genetic architectures that were difficult to dissect (Table S5). For example, the phenotypic variation explained by the QTLs for HSW ranged from 0.01% for *C3P33* in 2MN81 to 5.66% for C3P39 in MS2001_03, and for RPL ranged from 0.0001% for C13P45 in MS989 to 3.26% for *C5P34* in MAINT2016CR (Table S5). The identification of a large number of environmental- or populations-specific QTLs suggests that multiple effectors such as genetic background and environmental conditions influence the detection of QTLs for both traits in soybean.

Additionally, the majority of the associated SNPs in the QTLs for both traits exhibited a low minor allele frequency (<0.1) (Figure 2B,C), indicating positive selections acting on the major alleles corresponding to these QTLs. Only a few associated SNPs had a high minor allele frequency (0.1 < MAF < 0.5) (Figure 2B,C), which could be attributed to balancing selection or local adaptation.

### 2.3. Promising Candidate Genes for HSW QTLs

**Figure 3:**
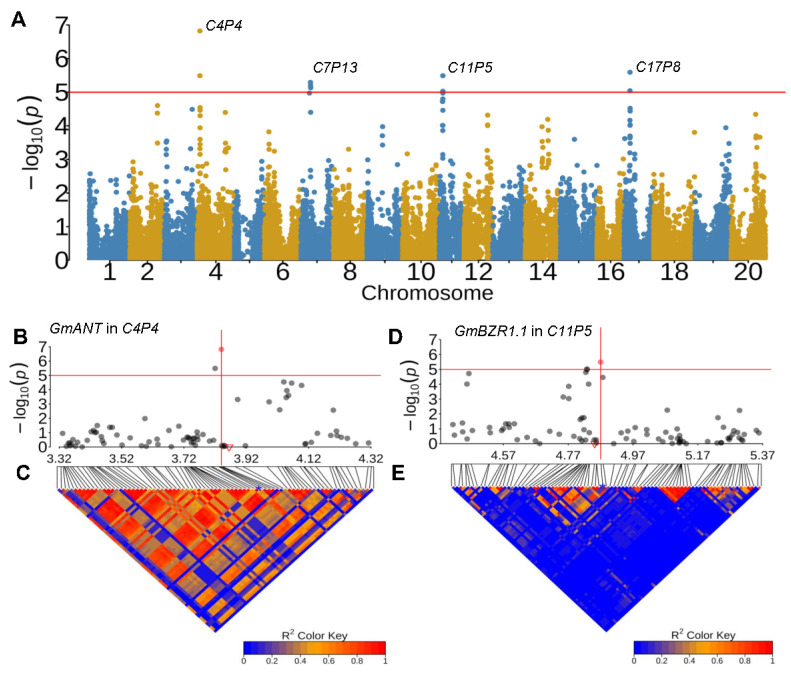
Genome-wide associations for 100-seed weight (HSW) in soybean. (**A**) Genome-wide association study for 100-seed weight using the phenotype from phenotype method: IL0102. The x-axis represents the position on 20 chromosomes, the y-axis represents the −log(*p*), and the red line represents the threshold to determine significant associations. (**B**) The regional association (±500 kb from leading SNPs: ss715587882) for *C4P4*. The red triangle represents the physical location of the candidate gene *GmANT*; the red solid dot represents the leading SNP. (**C**) LD heatmap of the association region corresponding to the region in (**B**). (**D**) The regional association (±500 kb from leading SNPs: ss715610794) for *C11P5*. The red triangle represents the candidate gene *GmBZR1.1*. (**E**) LD heatmap of the association region harboring *C11P5*. The red vertical lines in (**B**,**D**) indicate the physical location of the leading SNPs; the red solid points in (**B**,**D**) represent the leading SNPs; and the blue stars in (**C**,**E**) indicate the location of the leading SNP in the LD heatmaps.

In the present study, we applied an ortholog-co-localization approach to prioritize candidate genes for HSW QTLs. We began by retrieving 40 genes from *Arabidopsis* known to be associated with seed weight, functioning in either maternal or zygotic tissues [11]. Using the 40 proteins as queries, we identified a total of 125 orthologs in the soybean genome through protein BLAST with the *blastall* tool. The 125 putative seed weight regulatory genes, along with their chromosomal coordinates and Arabidopsis orthologs, are listed in Table S4 [11]. Among the 125 putative seed weight orthologs, eleven (8.8%) were found to be physically close to the HSW QTLs (within ±200 kb from the leading SNPs) (Figure 3; Table S5). Fisher’s exact test showed that the distribution of the eleven orthologs on the genome significantly differed (*p* < 0.001) from random distribution within the HSW QTLs, implying that these putative seed-weight-related genes are preferentially located within HSW QTLs. Hence, these soybean orthologs likely play similar roles in the regulation of seed weight, making them the most promising candidate genes for the corresponding HSW QTLs.

To further access the candidacy of the eleven orthologs for HSW QTLs, we analyzed regional linkage disequilibrium (LD) harboring the leading SNP for each HSW QTL. Remarkably, ten (90.9%) of the eleven genes were localized within the LD blocks containing the leading SNPs, further supporting that these orthologous genes are the candidate genes for coincident HSW QTLs. For example, *AtTTG2* (Transparent Testa Glabrous 2) in *Arabidopsis* regulates cell expansion in the maternal seed coat to influence seed size [32]. In soybean, two *TTG2* orthologs (*Glyma.03G176600* and *Glyma.19G177400*) were identified, both residing within the HSW QTLs (Table S5). Specifically, *GmTTG2.1* (*Glyma.03G176600*, Chr03:38,951,655–38,961,330) was physically located within the LD block carrying the leading SNP (ss715586034, Chr03: 39,134,285, *p* = 6.07 × 10^−07^) of QTL *C3P39* (Figure S1a,b; Table S5); while *GmTTG2.2* (*Glyma.19G177400*, Chr19: 43,672,864–43,681,647) resided under the association of QTL *C19P44* and it was located within the LD block harboring the leading SNP (ss715635294, Chr19: 43,670,133, *p* = 1.98 × 10^−06^) (Figure S1c,d; Table S5). Similarly, *AtANT* encodes an AP2-like family transcription factor and regulates organ growth and cell numbers during organogenesis in *Arabidopsis* [33,34]. One of the soybean orthologs, *GmANT* (*Glyma.04G047900*, Chr04: 3,858,051–3,862,348), was located within the LD block carrying the leading SNP (ss715587882, Chr04: 3,835,527, *p* = 1.50 × 10^−07^) of QTL *C4P4* on chromosome 4 (Figure 3B,C; Table S5). Furthermore, *AtBZR1*, encoding a transcription factor, regulates seed size by modulating seed size genes such as *SHB1*, *IKU1*, and *IKU2* [35,36]. Two homolog genes in soybean, *GmBZR1.1* and *GmBZR1.2* (*Glyma.11G064300* and *Glyma.14G076900*), were identified under the QTLs *C11P5* and *C14P6*, respectively. Furthermore, *GmBZR1.1* (Chr11: 4,849,798–4,851,673) was also situated within the LD block containing the leading SNP (ss715610794, Chr11: 4,869,122, *p* = 3.23 × 10^−06^) of QTL *C11P5* (Figure 3D,E; Table S5).

For QTL *C4P49*, *GmBRI1* (*Glyma.04G218300*, Chr04: 48,926,007–48,930,251), the ortholog of *AtBRI1* regulating seed size and seed shape in *Arabidopsis*, was identified under the association *C4P49*. Importantly, it carried the leading SNP (ss715588658, Chr04: 48,924,305, *p* = 5.85 × 10^−06^) (Figure S2a,b; Table S5), suggesting its candidacy for QTL C4P49. GmKLU (Glyma.06G310800, Chr06: 49,935,879–49,942,031), encoding the cytochrome P450 protein CYP78A5, is an ortholog of AtKLU, positively regulating seed size by promoting cell proliferation in the integuments [15,37]. It is regarded as one of the best candidate genes of QTL C6P50 (leading SNP ss715595178, Chr06: 50,377,967, p = 5.41 × 10^−07^) (Figure S2c,d; Table S5). GmABA2 (Glyma.19G197200, Chr19: 45,433,258–45,434,500) is an ortholog of AtABA2 that negatively regulates seed weight [38], and it was identified under the association (ss715635425, Chr19: 45,204,441, p = 6.71 × 10^−07^) of C19P45; hence, it is regarded as a candidate gene for QTL C19P45 (Figure S3c,d; Table S5). Similarly, on the same chromosome, GmABI5 (Glyma.19G194500, Chr19:45,201,611–45,206,816) was colocalized with QTL C19P45. GmABI5, an ortholog of AtABI5, can increase seed size by regulating ABA signal in Arabidopsis [38]. (Figure S3a,b; Table S5). These genes represent important targets for investigating the molecular mechanism underlying HSW in soybean.

### 2.4. Promising Candidate Genes for RPL QTLs

**Figure 4:**
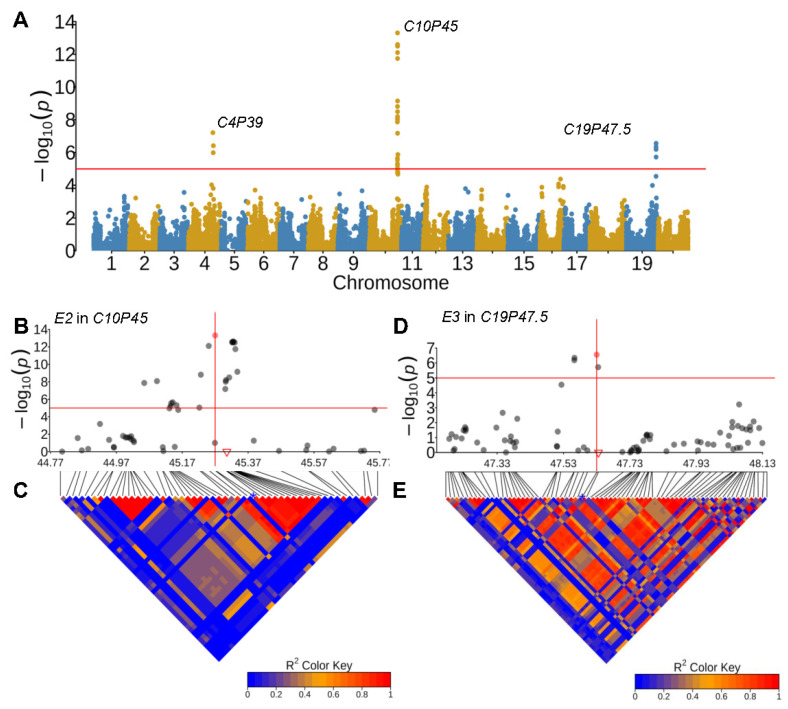
Genome-wide associations for reproductive period length (RPL) in *G. max*. (**A**) Genome-wide association study for reproductive period length using phenotype in 1IL66. The x-axis represents the position on 20 chromosomes, the y-axis represents the −log(*p*), and the red line represents the threshold to determine significant associations. (**B**) The regional association (±500 kb from leading SNPs: ss715607475) of *C10P45*, with the red triangle representing the candidate *E2*. (**C**) LD heatmap of the regional association region for *E2*. (**D**) The regional association (±500 kb from leading SNPs: ss715635703) of *C19P47.5*, with the red triangle representing the candidate *E3*. (**E**) LD heatmap of the association region for *C19P47.5*. The red solid points in (**B**,**D**) represent the leading SNPs, while the blue stars in (**C**,**E**) represent the location of the leading SNP in the LD heatmaps.

Reproductive period length (RPL) has been demonstrated to be closely associated with flowering time in plants. Among the 74 QTLs associated with RPL, six QTLs (*C10P45*, *C19P47*, *C11P11*, *C12P6*, *C20P34*, and *C16P31*) were found to be co-localized with or physically close to reported soybean flowering-time-controlling genes, including *E2*, *E3*, *Tof11*, *Tof12*, *E4*, and *FT2b* (Table S5). Of these six reported flowering-time-controlling genes, three (*E3, Tof11*, *GmFT2b*) were located within the LD blocks that coincided with the corresponding leading SNPs of the QTLs (Figure 4D,E; Table S5), thereby making them the best candidates for controlling the QTLs for RPL. For example, *Tof11* (*Glyma.U034500*), encoding a homologous pseudo-response-regulator gene that controls flowering time and maturity by modulating *E1* expression [25], was found close to QTL *C11P11* (leading SNP ss715608788, Chr11: 10,877,247, *p* = 3.86 × 10^−07^). *Glyma.16G151000* (*GmFT2b*, Chr16: 31,148,828–31,151,842) was nearly co-localized with *C16P31* (ss715624394, Chr16: 31,359,859, *p* = 3.61 × 10^−06^). It encodes a phosphatidylethanolamine-binding protein family protein (PEBP) that regulates soybean flowering time in Arabidopsis [39] (Table S5). QTL *C10P45* was identified in four environments (1IL66, 2IL811, IL0102, IL945), and the corresponding leading SNPs (ss715607475, Chr10: 45,269,968, *p* = 4.95 × 10^−14^; ss715607486, Chr10: 45,325,872, *p* = 3.13 × 10^−07^; ss715607495, Chr10: 45,463,820, *p*= 5.85 × 10^−07^; ss715607471, Chr10: 45,250,482, *p* = 2.04 × 10^−08^ were physically close to *E2* (*Glyma.10G221500*, Chr10: 45,294,734-45,316,121), a major flowering controlling gene in soybean [29] (Figure 4B,C; Table S5). *E3* (*Glyma.19G224200*, Chr19: 47,633,058–47,660,246) encodes phytochrome protein (GmPHYA3) function in post-flowering photoperiod response [40], and it overlapped the leading SNP (ss715635703, Chr19: 47,632,093, *p* = 2.76 × 10^−07^) for QTL *C19P47* (Figure 4D,E; Table S5). The precise identification of these flowering-time- and photoperiod-sensitive genes in RPL-associated QTL regions indicates the robustness of our analysis, suggesting that these flowering-time-controlling genes were likely involved in the regulation of RPL. These promising results encourage further investigation into candidate genes for other QTLs.

**Figure 5:**
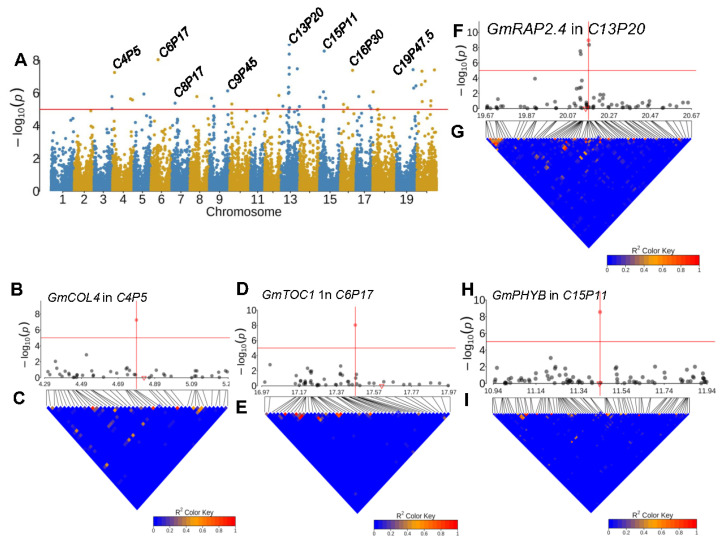
Genome-wide associations for reproductive period length in *G. soja*. (**A**) Genome-wide association study for reproductive period length using phenotype from MS989. (**B**) The regional association (±500 kb from leading SNPs: ss715588860) of *C4P5*, with the red triangle denoting the candidate *GmCOL4*. (**C**) The LD heatmap of the association region for *C4P5*, with the blue star representing the location of the leading SNP. (**D**) The regional association (±500 kb from leading SNPs: ss715593757) of *C6P17*, with the red triangle indicating the candidate *GmTOC1*. (**E**) The LD heatmap of associational region for *C6P17*, with the blue star representing the leading SNP of *C6P17*. (**F**) The regional association (±500 kb from leading SNPs: ss715613915) of *C13P20*, with the red triangle representing the candidate *GmRAP2.4*. (**G**) The LD heatmap of associational region for *C13P20*, with the blue star representing the leading SNP of *C13P20*. (**H**) The regional association (±500 kb from leading SNPs: ss715620326) for *C15P11*, with the red triangle indicating the candidate *GmPHYB*. (**I**) The LD heatmap containing the association region for *C15P11*, with the blue star indicating the leading SNP of *C15P11*. The red solid points in (**B**,**D**) represent the leading SNPs, and the blue stars in (**C**,**E**) indicate the locations of the leading SNPs in the LD heatmaps.

Using the same strategy as mentioned above, we also identified candidate genes for an additional 13 RPL QTL regions; these genes have been reported to be involved in the regulation of flowering time or photoperiod sensitivity in *Arabidopsis* but have yet been characterized in soybean (Figure 5; Table S5). CONSTANS acts to induce the photoperiodic flowering of *Arabidopsis* [41]. Several of its homologs in soybean were identified as candidate genes for several RPL QTLs (Table S5). For example, *Glyma.04G058900* (*GmCOL4*, Chr04: 4,830,133–4,831,900) was an ortholog of *AT5G24930* (*COL4*), which is a flowering repressor in long days and short days by regulating the expression of *FT* and *FT*-like genes [42]. It was colocalized with *C4P5* with leading SNP: ss715588860 (Chr04: 4,789,744, *p* = 5.63 × 10^−08^) (Figure 5B,C; Table S5); *AtCO5* encodes a *CONSTANS-LIKE 5* gene, which regulates flowering time under short days [43]. *Glyma.13G093800* (*GmCO5*, Chr13: 20,903,248–20,905,141) is an ortholog of *AT5G57660* (*AtCO5*), and it colocalized with QTL *C13P21* with the leading SNP ss715617283 (Chr13: 20,939,432, *p* = 8.11 × 10^−06^) (Table S5).

*AGAMOUS-LIKE (AGL)* family genes have been reported to play roles in the regulation of flowering time, and soybean *AGL* genes were also identified as potential candidate genes for RPL (Table S5). For example, *Glyma.04G159300* (*GmAGL8*, Chr04: 39,283,911–39,295,261) is an ortholog of *AT5G60910*, a suppressor of axillary meristem maturation that promotes longevity in flowering plants [44], and it was colocalized with *C4P39* (leading SNP ss715587808, Chr04: 39,006,019, *p* = 6.13 × 10^−08^) (Table S5). *Glyma.14G019400* (*GmAGL5*, Chr14: 1,381,763–1,396,383) is a homolog of *AT2G42830* encoding a K-box region and MADS-box transcription factor family protein, playing a role in flowering time and maturity [45]. *GmAGL5* was colocalized with QTL *C14P1* (leading SNP ss715617711, Chr14: 1,361,184, *p* = 4.30 × 10^−06^). Additionally, two *AGL9* homologous genes in soybean were identified as the candidate genes underlying RPL QTLs. *Glyma.05G148800* (*GmAGL9.1*, Chr05: 34,293,638–34,302,134) is a member of the MADs box transcription factor family gene, and it was colocalized with QTL *C5P34* (leading SNP ss715591020, Chr05: 34,493,210, *p* = 7.15 × 10^−08^) (Table S5). Its *Arabidopsis* homolog *AT1G24260* is involved in initiation and development of flowers [46,47]. The other *AGL9* ortholog gene, *Glyma.10G240900* (*GmAGL9.2*, Chr10: 46,928,296–46,933,075), was colocalized with RPL QTL *C10P47* (leading SNP ss715607613, Chr10: 46,706,603, *p* = 1.46 × 10^−06^) (Table S5); thus, it was regarded as a promising candidate gene for the QTL.

In addition to *Tof11* and *Tof12*, which encode PSEUDO-RESPONSE REGULATOR 1 (PRR1), another candidate gene for RPL was identified (Table S5). We found a homolog of *AT5G61380* (*AtPRR1*), also known as *TOC1*, to be a potential candidate gene for an RPL QTL. For example, *AtPRR1* is known for its role in regulating plant flowering time in response to photoperiod [48]. One of its orthologs *Glyma.06G196200* (*GmTOC1*, Chr06: 17,608,368–17,614,484) was found to be colocalized with *C6P17* (leading SNP ss715593757, Chr06: 17,470,006, *p* = 9.30 × 10^−09^) (Figure 5D,E; Table S5).

Moreover, several genes encoding phytochrome proteins, whose Arabidopsis homologs play roles in modulating flowering through perceiving light signals, were also identified within the RPL QTL regions (Table S5). For example, *AT4G18130* encodes phytochrome E, which plays an important role in regulating the initiation of flowers [49]. *AT2G18790* encodes a phytochrome B protein (PHYB), and mutation in *PHYB* promotes flowering time during both long and short days (www.arabidopsis.org). Its ortholog *Glyma.09G088500* (*GmPhyE*, Chr09: 11,694,596–11,700,954) was close to RPL QTL *C9P11* (leading SNP ss715602973, Chr09: 11,217,463, *p* = 6.47 × 10^−06^) (Table S5). Likewise, another homolog of *PHYB*, *Glyma.15G140000* (Chr15: 11,435,550–11,442,683), was colocalized with *C15P11* (leading SNP ss715620326, Chr15: 11,441,207, *p* = 2.78 × 10^−09^) (Figure 5H,I; Table S5). Additionally, the Phytochrome Interacting Factor 3 (PIF3) (*AT1G09530*) gene could modulate flowering time by responding to light. In soybean, two *PIF3* genes (*Glyma.19G222000*, Chr19: 47,396,908–47,399,851 and *Glyma.19G224700*, Chr19: 47,691,500-47,696,511), were identified within the same LD block containing *E3* (Figure 4D,E; Table S5), implying that the two PIF3s were also the candidate genes for QTL *C19P47*. *AT1G78080* regulates flowering time and cotyledon development by mediating light and ethylene signaling [50]. Its ortholog, *Glyma.13G088100* (*GmRAP2.4*, Chr13: 20,155,570–20,157,489), was colocalized with *C13P20* (leading SNP ss715613915, Chr13: 20,169,935, *p* = 1.11 × 10^−09^) (Figure 5F,G; Table S5).

Other soybean genes with the Arabidopsis homologs functioning in flowering control were also identified and regarded as RPL QTL candidate genes because they coincided with the corresponding QTL (Table S5). For example, a gene family of cytochrome P450 was identified to be involved in plant maturation [51], and a homolog of flower-specific expressed gene *AT5G52400* (*CYP715A1*), *Glyma.09G137600* (*GmCYP715A1*, Chr09: 34,094,418–34,098,004), was physically close to RPL QTL *C9P34* (leading SNP ss715603584, Chr09: 34,103,263, *p* = 3.89 × 10^−06^) (Table S5). *AT4G36920* (*AP2*) codes for an integrase-type DNA-binding superfamily protein and regulates the termination of apical meristem at various stages, both vegetative and reproductive [52,53]. One of its homologs in soybean, *Glyma.11G053800* (*GmFLO2*, Chr11: 4,048,454–4,052,090), was located near QTL *C11P4* (leading SNP ss715610662, Chr11: 3,997,743, *p* = 5.02 × 10^−06^) (Table S5). The function of the two genes has not been investigated in soybean, thus warranting further investigation into their function in soybean.

### 2.5. Allele Distribution of the QTLs Altered during Domestication and Improvement

**Figure 6:**
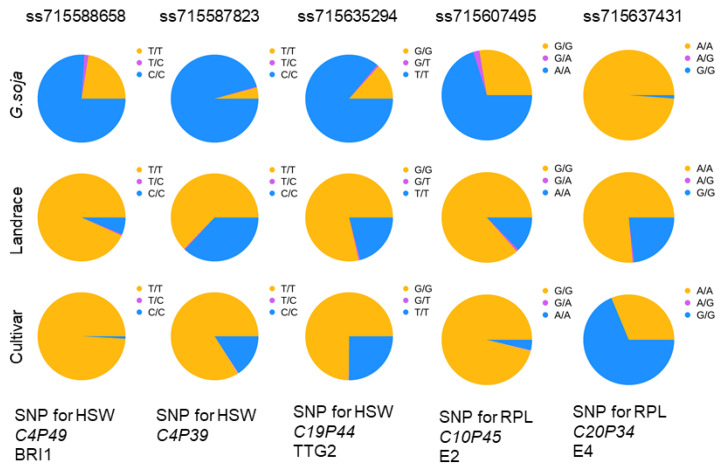
Allele frequencies for the leading SNPs ss715588658, ss715587823, and ss715635294 for HSW, and ss715607495, and ss715637431 for RPL in *G. soja*, landrace, and cultivar.

HSW and RPL are two major domesticated traits in soybean subjected to artificial selection, as evidenced by the significant differences between *G. soja* and *G. max* (Figure 1A,B). These dramatic differences in the traits promote us to examine whether the identified QTLs were targeted during soybean domestication or breeding. To address this, we divided the population into three germplasm types, wild (*G. soja*), landrace (*G. max*), and cultivar (*G. max*). Allele frequency analysis revealed varying changes in the allele distribution of some leading SNPs in the QTLs for HSW or RPL across the three subpopulations (Figure 6). For example, ss715588658 (*p* = 5.85 × 10^−06^), the leading SNP of HSW QTL *C4P49*) with the nominated candidate gene *GmBRI1*, showed notable differences in allele distribution. The allele C predominated (76%) in *G. soja*, while the alternative allele T was predominant (94%) in landrace and nearly fixed (99.2%) in cultivar plants (Figure 6). Similarly, for the leading SNP ss715587823 (*p* = 2.07 × 10^−06^) for QTL *C4P4* for HSW, the allele C was the major allele (96%) in *G. soja*, but its frequency decreased to 37% in landrace and 16% in cultivar. This suggests a continuous increase in the frequency of the alternative allele T during the transition from *G. soja* to landrace and cultivar. A similar pattern of increased allele frequency was also observed for ss715588658 (*GmBRI1*), indicating successive selection of the QTL region or the candidate genes, likely for larger seed size (Figure 6). Another example is ss715635294 (*p* = 1.98 × 10^−06^) for HSW with candidate *GmTTG2*. In this case, allele T was the dominant allele (86%) in *G. soja*, while the alternative allele G became the major allele in *G. max*, with comparable frequencies between landrace and cultivar (79% in landrace and 75% in cultivar) (Figure 6). This suggests that this allele underwent selection during domestication but not improvement.

Similar patterns of frequency shifts between the subpopulations were also observed for some associations for RPL. For example, for the leading SNP ss715637431 for QTL (*C10P45*, *p* = 3.02 × 10^−06^) containing *E4*, the allele A is predominant (99%) in *G. soja*, while frequency of the alternative allele G was continuously increased during domestication (23% in landrace) and breeding process (69% in cultivar) (Figure 6). Similarly, for the leading SNP ss715607495 (*p* = 5.85 × 10^−07^) for QTL *C10P45* harboring *E2*, A is the major allele (70%) in *G. soja*, while its frequency was significantly decreased during domestication (13% in landrace) and the breeding process (4% in cultivar) (Figure 6). This suggests that the alternative allele G was targeted during both domestication and improvement. The dominant allele *E2* is prevalent in *G. soja* and can delay flowering, while the recessive allele *e2* can promote flowering, likely favoring longer RPL and increased soybean yield in cultivated soybean [54,55]. These results suggest that alleles with high frequency in cultivated soybean significantly contribute to the desired RPL for soybeans and were consistently selected during the improvement.

### 2.6. RPL Is Correlated with Geographic Adaptation

**Figure 7:**
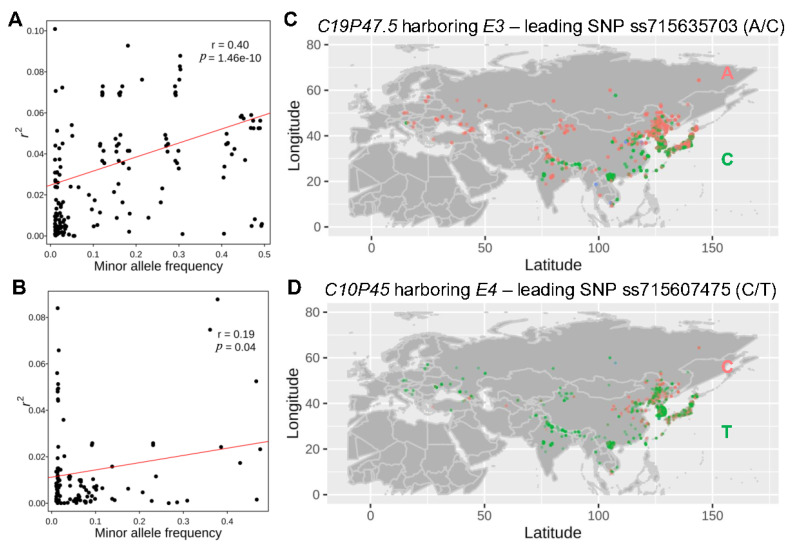
The geographic distribution of alleles for significant associations. (**A**) The relationship between the correlation of association with latitude and the minor allele frequency. (**B**) The relationship between the correlation of associations with longitude and minor allele frequency. (**C**) The geographic distribution of alleles for *E3* using associated SNPs ss715635703 for QTL and *C19P47.5* for RPL. Red dots represent allele “A”, green dots represent allele “C”, and blue indicates heterozygosity “Y”. (**D**) The geographic distribution of allele for *E2* uses associated SNPs ss715607475 for *C10P45* for RPL. Red dots represent allele “C”, green dots represent allele “T”, and blue indicates for heterozygosity “Y”.

Soybean is known before its photoperiod sensitivity, requiring specific cultivation in distinct geographic regions to ensure optimal maturation and productivity. To access whether certain QTLs for both RPL and HSW had underwent artificial selection, we employed a regression model to explore the relationship between latitude and the geographic distribution of associated leading SNPs for QTLs. For RPL, we observed a significant relation (*r*^2^) between the geographic distribution of associated SNPs and latitude, ranging from 0 to 0.1 (Figure 7A). Given the diversity of leading SNPs for QTLs (Figure 2B,C), we investigated the correlation between the relation (*r*^2^) of the associated SNPs with geographic location and their diversity (measured as minor allele frequency: MAF). This analysis aimed to elucidate the relationship between SNP selection and geographic adaptation. Our results revealed a significant correlation between MAF and the correlation of RPL QTLs with latitude (*r* = 0.40; *p* = 1.46 × 10^−10^) (Figure 7A), but not for longitude (*r* = −0.11, *p* = 0.09). This suggests that the latitude adaptation driven by human selection likely plays a crucial role in diversifying some SNPs associated with RPL, and SNPs with distinct geographic distribution patterns likely contributing to local adaptation, including RPL. For example, *E3*, a photoperiod-sensitive response gene [40], is the candidate gene for RPL QTL *C19P47.5*. The leading SNP (ss715635703) for this QTL exhibited clear geographic distribution of E3 (Figure 4D and Figure 7C), with one allele (A) for *E3* highly enriched in the northern or highland regions, while the alternative allele (C) is preferentially distributed in the southern area (Figure 7C). The strong correlation between the leading SNP (ss715635703) for QTL *C19P47.5* and latitude distribution underscores the importance of the photoperiod-sensitive response gene *E3* in soybean latitude-based geographic adaptation (Figure 7C). Similarly, the leading SNP (ss715607475) of QTL *C10P45* for flowering-time-controlling gene *E2* also showed that allele C was enriched in Northeast Asia, while the alternative allele T was mixed with allele C in Northeast Asia and South Asia and at high altitudes (Figure 7D). These results validate that RPL is an adaptation trait that is regulated, in part, by flowering-time-controlling genes, such as *E2* and *E3*, and further suggest that domestication and improvement processes might contribute to higher genetic diversity at these loci for better geographic adaptation.

Similar to RPL, a correlation between MAF and the correlation of the associated SNP for HSW with latitude was also observed. However, the correlation was marginally significant (*r* = 0.19, *p* = 0.04) (Figure 7B), while no correlation was observed with longitude for HSW (*r* = 0.17, *p* = 0.06). This result suggests that the geographic location had an effect, albeit small, on the diversification of HSW-associated SNPs.

## 3. Discussion

### 3.1. Homolog-Guided GWAS: An Effective Strategy for Prioritizing Candidate Genes

Seed size and reproductive period are major yield-related traits and have been targeted for improvement in soybean. However, both traits are quantitatively controlled by numerous genes, the majority of which have small effects. It has been reported that over 300 QTLs play roles in the regulation of HSW and RPL (https://www.soybase.org/). Despite some progress in uncovering controlling genes such as *SWEET39*/*SWEET10a* [26,27] and *GmST05* [17], the underlying mechanism for either trait remains largely unknown. Therefore, identifying the genes controlling HSW or RPL has become a primary goal of fundamental research in soybean.

Identifying the molecular basis of seed size in soybeans has been challenging due to the effects of genetic background and environments, and few seed-size-regulating genes have been identified using traditional approaches, such as fine-mapping-based QTL cloning, which is time-consuming and labor-intensive. GWAS is a prevalent approach that is widely used to understand the genetic basis of natural variation [56,57], especially for the traits controlled by multiple genes, such as flowering time [58]. In this study, we applied the GWAS approach to dissect the genetic architecture of HSW and RPL using multiple subsets of accessions from GRIN. This approach accounts for effects from genetic background or environments, allowing for the maximum capture of genomic variation underlying both traits. Indeed, we identified 61 QTLs for HSW and 74 QTLs for RPL, most of which were specific to certain individual populations or environments. Further determination of the candidate genes underlying these QTLs, as uncovered in our study, would provide molecular comprehension of how environment variation affects phenotypic expression. Using phenotypes collected from different subsets of the GRIN germplasm under different environments for GWAS would facilitate the identification of association heterogeneous loci [59,60] that might be overshadowed in individual populations or under a certain environment. All the QTLs identified in our GWAS explained relatively low phenotypic variation, suggesting that phenotypic variation in both traits results from the combined effects of many genes with small effects, which might be the major challenge in uncovering candidate genes. Nevertheless, with the large-scale GWAS, we were able to dissect the ever-complex architecture of HSW and RPL.

Here, we demonstrated that a comparative genomics-based approach is a valuable method for prioritizing high-confidence candidate genes by integrating homologs with relevant roles into the genetic mapping result in soybean [12,56]. This strategy was motivated by evidence showing that many genes controlling development, such as flowering time, were conserved between soybean and Arabidopsis. Examples include seed-filling-associated genes *SWEET39*/*SWEET10a* [26,27], *PRR* genes, *ELF3*, regulating flowering times [24,25], and *CYP78A*, influencing seed weight [13,15]. The identification of previously reported genes involved in flowering time and HSW in Arabidopsis within the QTL region in soybean demonstrates the robustness of the gene discovery approach. The enrichment of these *Arabidopsis* orthologs regulating seed size, flowering time, and photoperiod response genes in HSW and RPL QTL regions further confirms conserved regulatory mechanisms and genetic basis of seed size and flowering control between soybean and *Arabidopsis* [11,61,62]. This result also suggests that the genetic architecture and regulatory mechanisms of seed weight and RPL/flowering are ancient mechanisms that have been partially maintained in two divergent species, likely due to the critical role of these genes/mechanism in producing viable seeds. It has been reported that RPL negatively correlates with flowering [18,19], and our study provides genetic evidence that many shared flowering genes may contribute to their correlation. Recent studies have revealed a sequential regulatory pathway of *E3-Tof11/Tof12*-*E1*-*FT2b* in the regulation of flowering [25], and other genes, like *CO* and *AGL* genes, were also involved in the controlling flowering time in soybean [63,64,65]. This may partially explain a previous study where reproductive period length was associated with the photoperiod [66]. How this gene expression cassette molecularly regulates RPL deserves experimental determination. Therefore, the results demonstrate the effectiveness of homolog-guided GWAS in prioritizing candidate genes. Despite several prioritized genes, candidate genes underlying other QTLs remain to be identified.

### 3.2. RPL as a Domestication Trait Selected for Enhanced Soybean Yield

In most seed crops, seed size and reproductive period are major yield-related traits subject to artificial selection [54]. It has been demonstrated that seed size significantly increased during the domestication process in cereal crops [67], and the same holds true for soybean [26,27,68]. It is reasonable to infer that RPL was substantially prolonged in *G. max* compared to *G. soja*, as a longer reproductive period generally yields larger seeds favored by humans and increased seed number per plant [20,66,69,70]. The transition from a lack of correlation between HSW and RPL in *G. soja* to a positive correlation in *G. max* suggests that artificial selection contributes to this correlation because selection for larger seeds and longer RPL in *G. max* aligns in the same direction, likely aiming for better yields. In contrast, *G. soja* accessions are shorter in reproductive period than *G. max*. This shorter period likely enhances the fitness of *G. soja* by enabling it to swiftly complete its life cycle to produce viable seeds for the next generation. In addition, we further demonstrated that many of the HSW and RPL QTLs were targeted during the selection for longer RPL or larger seeds, indicating that both traits were domestication traits. These findings have been scarcely reported previously, mainly due to a lack of QTLs or candidate genes for both traits. Therefore, our study suggests that identifying candidate genes for both traits aids in understanding how both traits were selected from genetic perspective. Importantly, some of the QTLs are currently undergoing selection in contemporary soybean breeding programs, highlighting their importance in the present-day and future soybean improvement for enhanced HSW and RPL.

### 3.3. Local Adaptation Contributes to the High Diversity of RPL QTLs

It is expected that favored alleles would undergo higher selection pressure than neutral alleles, and intense selection for favored alleles would consequently lead to a reduction in minor allele frequency or diversity. We demonstrated that both RPL and HSW were domestication traits subject to artificial selection. Despite reduced minor allele frequencies observed for most identified QTLs, some associations exhibited relatively high minor allele frequency (Figure 2B,C). The significant correlation between the diversity of associated SNPs and latitude (Figure 7A) suggests that the high diversity of certain associated SNPs may stem from local adaptation due to artificial selection, rather than balancing selection. Since different latitudes or day lengths may impose distinct selection pressures [71], different alleles would be favored at different latitudes or day lengths for local adaptation [72]. However, in specific populations from various ecological regions, diversity may be low in related loci due to local adaptation. Given that many RPL QTLs contribute to soybean adaptation, investigating these allelic variations or region-adaptive alleles of RPL QTLs in a large soybean population across different environments could elucidate how RPL was genetically controlled and selected for adaptation [24,25,73].

## 4. Materials and Methods

### 4.1. Phenotypic Data

The phenotypic data, including 100-seed weight (HSW, g), flowering date, and maturity date, were sourced from the Germplasm Resources Information Network of the USDA-ARS (GRIN, www.ars-grin.gov). HSW, flowering date, and maturity date were collected across various subsets of the USDA-Soybean Germplasm Collection spanning multiple environments (years and locations) (Table S1). Reproductive period length (RPL, days) per environment was calculated as the number of days between the recorded flowering date and the maturity date. The differences in HSW or RPL between wild soybean *G. soja* and cultivated soybean *G. max* were examined using the *t.test* function in R (https://www.rdocumentation.org/packages/stats/versions/3.6.2/topics/t.test, accessed on 19 February 2024). Phenotypic correlations between two traits were computed in different germplasms (*G. soja* and *G. max*) using the *cor.test* function in R (https://www.rdocumentation.org/packages/stats/versions/3.6.2/topics/cor.test, accessed on 19 February 2024).

### 4.2. Genotypic Data

All germplasms used in the present study have been genotyped using the SoySNP50K iSelect BeadChip, as described in previous studies [74,75]. The SNP data were obtained from the SoyBase database (http://www.soybase.org), and only SNPs identified on 20 chromosomes were used for analysis in this study.

### 4.3. Heritability

The heritability was calculated using the phenotypic data for a set of accessions that were grown in two distinct environments. Specifically, 250 accessions were used for HSW, and 249 accessions were used for RPL. These accessions were phenotyped in Urbana, Illinois, in 2001 and 2002 (IL0102), and Rosemount, Minnesota, in 2001 and 2002 (MN0102). Heritability was calculated using the variation components detected *lme4* package (version 1.1-35.1) [76]:

*h*^2^ = *Vg*/(*Vg* + *Ve*)

where *h*^2^ represents the broad sense heritability, *Vg* denotes the genetic variation, and *Ve* signifies the error.

Using the same dataset, the effects of genotype or environment were analyzed using a model:

*y_ij_* = *u* + *G_ij_* + *E_j_* + *e_ij_*

where *y_ij_* denotes the observation of genotype *I* under environment *j*, *G_ij_* represents the genotype *i* in environment *j*, *E_j_* signifies the environment *j* used for phenotype, and *e_ij_* stands for the error. The model was fitted using the *lm* function in R (https://www.rdocumentation.org/packages/stats/versions/3.6.2/topics/lm, accessed on 19 February 2024).

### 4.4. Genome-Wide Association Study

A genome-wide association study (GWAS) was performed using subsets of individuals collected in a specific environment (Table S1) to mitigate the environmental effect. Only populations containing greater than 200 accessions was used for GWAS. The linear mixed model was applied for GWAS using the Genome Association and Prediction Integrated Tool (GAPIT) with minor allele frequency > 0.01, the default setting for Kinship, and Model.selection = TRUE [77,78,79]. The threshold was set at a *p*-value of 10^−5^. Visualization of GWAS results was facilitated by drawing a Manhattan plot using *qqman* [80]. The linkage disequilibrium (LD) heatmap for the association regions of interest was drawn using the lDheatmap package [81], and the Manhattan plot for the regional association was generated using a customized R code of *qqman*. Associations were named based on the physical position of the identified QTL on the chromosome. For example, a QTL that was named *C11P11* signifies that the QTL (primarily the leading SNP) was situated or physically close, approximately 11 Mb region on chromosome 11. Regression between the allelic distribution of associated SNPs and the geographic origin of the germplasm (latitude or longitude) was performed using the *lm* function in R. The geographic map was plotted using R packages *rworldmap*, and *ggplot2* (https://www.rdocumentation.org/packages/rworldmap/versions/1.3-8, https://www.rdocumentation.org/packages/ggplot2/versions/3.4.4, accessed on 19 February 2024).

### 4.5. Identification of Candidate Genes

Candidate genes for the identified QTL were prioritized based on the colocalization of Arabidopsis genes associated with flowering, maturation, and seed size with the QTLs or within the same LD block harboring leading SNPs. *Arabidopsis* genes with known roles controlling seed size or flowering were selected from recent research articles or review papers [11]. Fisher’s exact tests were performed using R (https://www.rdocumentation.org/packages/stats/versions/3.6.2/topics/fisher.test, accessed on 19 February 2024) to access whether the orthologs were randomly distributed across the genome relative to our GWAS for HSW.

## 5. Conclusions

We identified 61 QTLs for HSW and 74 QTLs for RPL through large-scale GWAS in multiple populations with diverse backgrounds across various environments. Eleven and thirteen *Arabidopsis* orthologs in soybean and six soybean genes with known roles controlling seed weight and flowering time, respectively, were identified and nominated to be the best candidate genes for further investigation of HSW and RPL QTLs. Our results preliminarily demonstrate the shared genetic basis of HSW or RPL between divergent soybean and *Arabidopsis*. Most of the SNPs associated with RPL were correlated with geographic adaptation in soybean, suggesting that local adaptation shaped the diversity pattern at these loci, which could be emphasized in further research. One limitation of the study is that we prioritized gene discovery for HSW and RPL based on reported genes in Arabidopsis, potentially overlooking other genes within QTL or coincident LD regions. We have listed all identified QTLs and leading SNPs in the study for further investigation by the research community. Nevertheless, our study presents an effective approach for genome-wide elucidation of the genetic basis of complex traits, particularly for traits maintaining conserved regulatory mechanisms observed in well-studied Arabidopsis. Furthermore, the study identifies a set of candidate genes as a valuable resource for functional genomic research on HSW and RPL, offering insight into the genetic architecture of HSW and RPL for soybean seed weight and adaptation improvement.

## Data Availability

Data presented in this paper is contained within the article and Supplementary Materials.

## Supplementary Materials

The following supporting information can be downloaded at: https://www.mdpi.com/article/10.3390/plants13050615/s1, Figure S1. The regional association for C3P39 and C19P44. (a) The regional association for C3P39 and (b) the LD heatmap for the region of C3P39. (c) The regional association for C19P44 and (d) the LD heatmap for the region of C19P44. The red triangle represents the candidate gene. Figure S2. The regional association for C4P49 and C6P50. (a) The regional association for C4P49 and (b) the LD heatmap for the region of C4P49. (c) The regional association for C6P50 and (d) the LD heatmap for the region of C6P50. The red triangle represents the candidate gene. Figure S3. The regional association for C19P45 and C19P46. (a) The regional association for C19P45 and (b) the LD heatmap for the region of C19P45. (c) The regional association for C19P45 and (d) the LD heatmap for the region of C19P45. The red triangle represents the candidate gene. Table S1. The information for the environments and the abbreviations used in the study. Table S2. The Pearson correlation between hundred-seed weight and productive period length. Table S3. The correlation between traits and geographic locations. Table S4. Candidate genes for associations of HSW. Table S5. GWAS results for HSW and RPL and proposed candidate genes.
