# Supplementary material for: Genome-Wide Association Studies Prioritize Genes Controlling Seed Size and Reproductive Period Length in Soybean
Source: Plants (Basel). 2024 Feb 23;13(5):615. doi: 10.3390/plants13050615 (PMC10933924; doi:10.3390/plants13050615)
Supplement: Supplementary file 1 [file plants-13-00615-s001.zip › Supplimentary_Figures_R1.pdf]

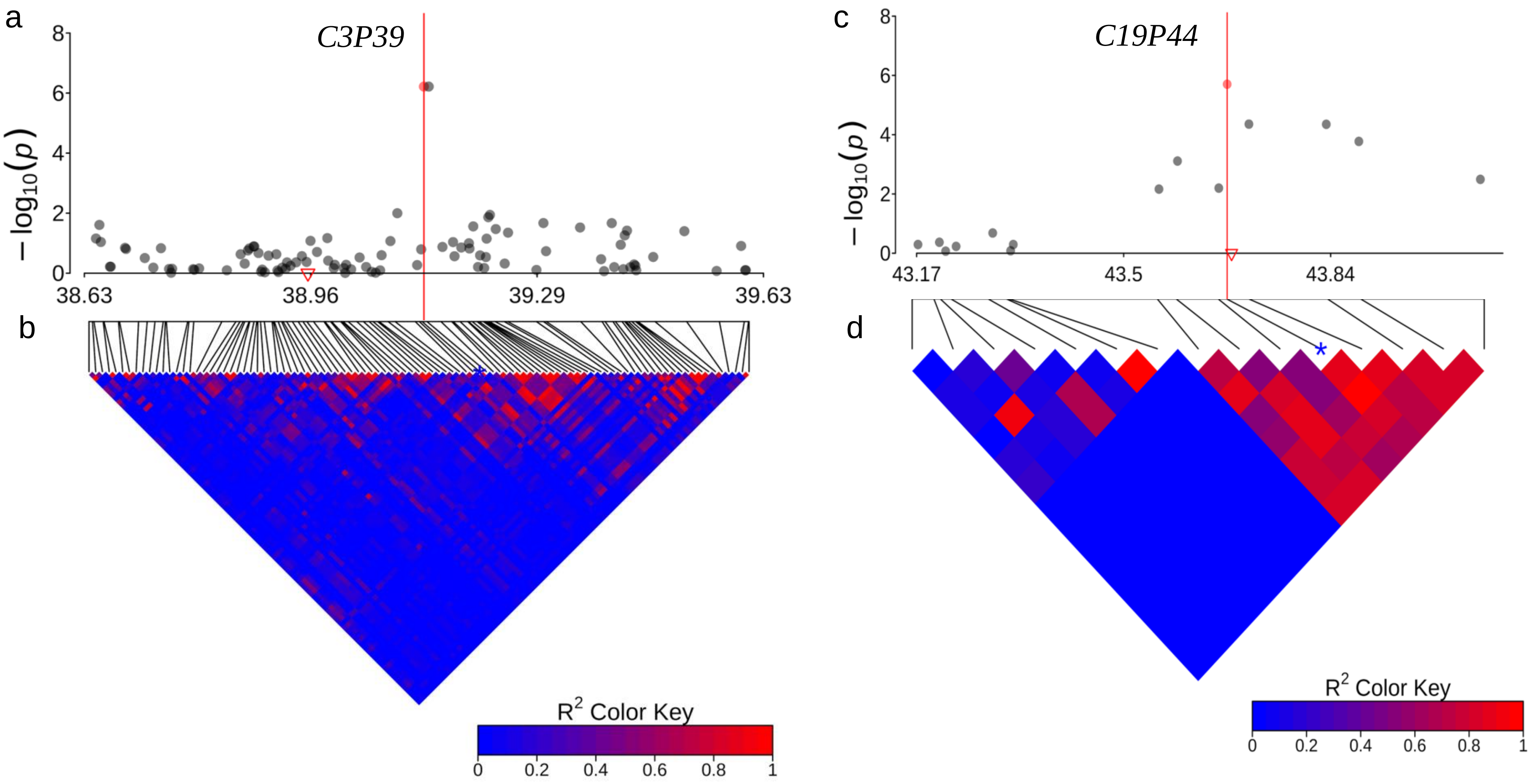

Figure S1 The regional association for *C3P39* and *C19P44*. a) The regional association for *C3P39* and b) the LD heatmap for the region of *C3P39*. c) The regional association for *C19P44* and d) the LD heatmap for the region of *C19P44*. the red triangle represents the candidate gene.

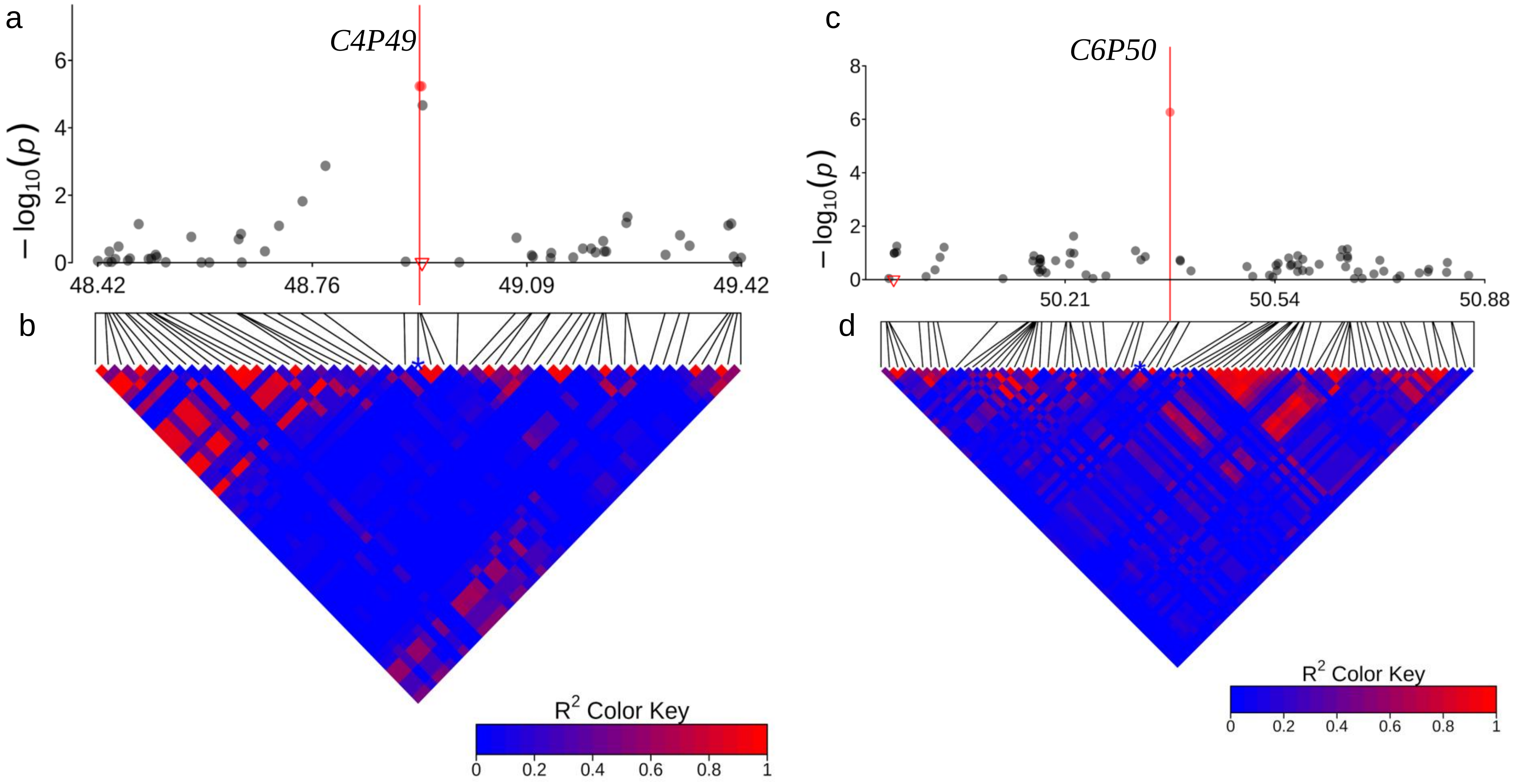

Figure S2 The regional association for *C4P49* and *C6P50*. a) The regional association for *C4P49* and b) the LD heatmap for the region of *C4P49*. c) The regional association for *C6P50* and d) the LD heatmap for the region of *C6P50*. The red triangle represents the candidate gene.

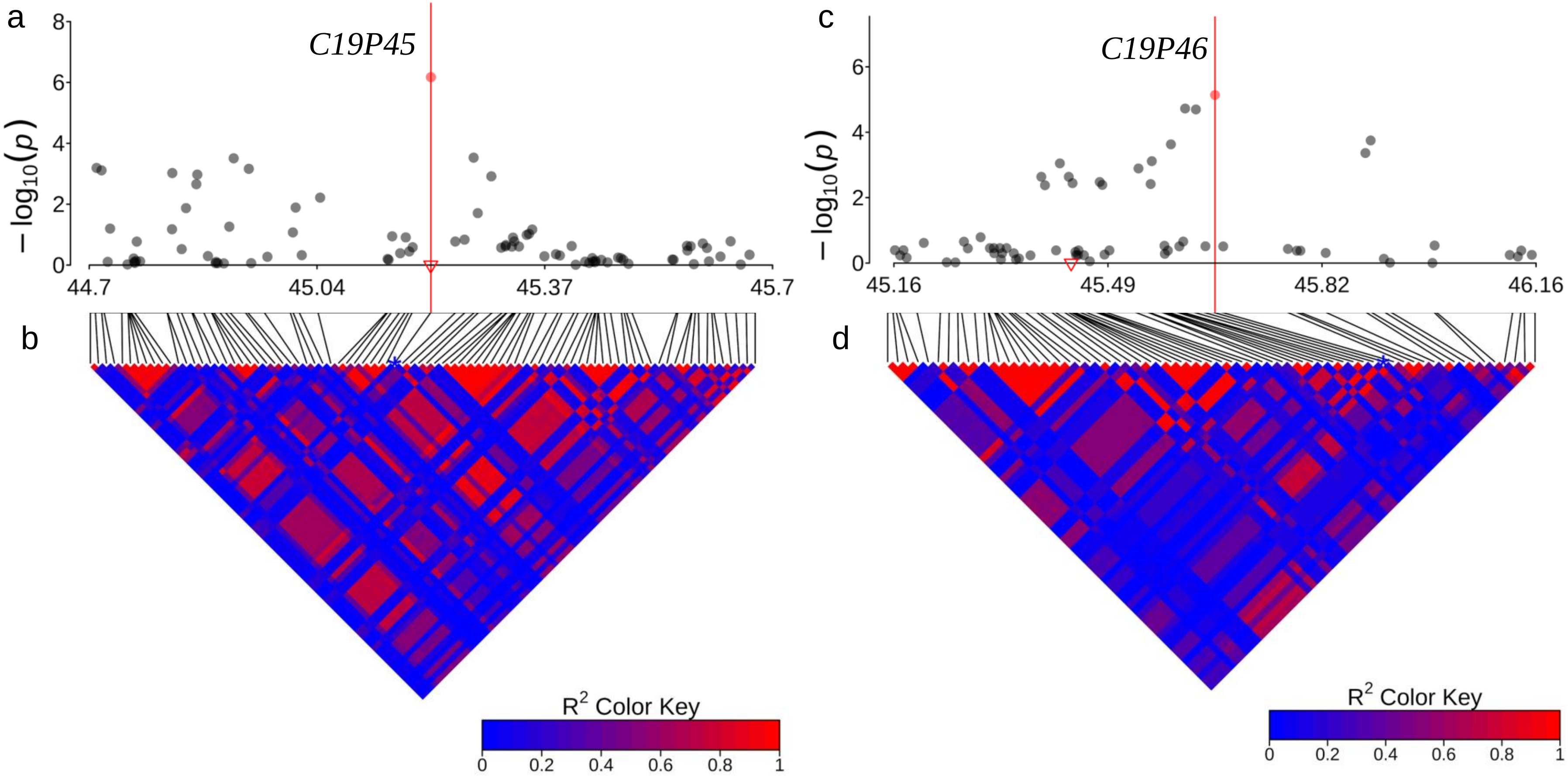

Figure S3 The regional association for *C19P45* and *C19P46*. a) The regional association for *C19P45* and b) the LD heatmap for the region of *C19P45*. c) The regional association for *C19P45* and d) the LD heatmap for the region of *C19P45*. The red triangle represents the candidate gene.
